# Spatiotemporal Dynamics of *Buddleja officinalis* Maxim. Habitat Suitability Under Climate Change in China

**DOI:** 10.3390/biology15211984

**Published:** 2026-11-02

**Authors:** Ying Liu, Wenyuan Chen, Fangfang Kong, Yueqin Leng, Yan Yang, Yangzhou Xiang

**Affiliations:** 1 School of Biological Sciences, Guizhou Education University, Guiyang 550018, China; 2 Department of Pharmacy, Bijie Medical College, Bijie 551700, China; 3 School of Geography and Resources, Guizhou Education University, Guiyang 550018, China

**Keywords:** *Buddleja officinalis*, MaxEnt model, climate change, medicinal plant, suitable habitat, species distribution

## Abstract

The geographical distribution of plant species is being fundamentally altered by ongoing global climate change. *B. officinalis*, an important medicinal and nectar plant native to South China, may experience uncertain alterations in its suitable habitat under projected climatic changes. To predict where *B. officinalis* may find suitable environments across China, this work drew upon 346 presence records alongside 24 predictor layers (climatic, topographic, vegetative, and anthropogenic) and employed an ENMeval-tuned MaxEnt framework. Habitat suitability was projected for the present day and three future time slices (2050s, 2070s, 2090s) under four Shared Socioeconomic Pathways (SSP126–SSP585). The main results are as follows. (1) Bio7 (temperature annual range), altitude, and Bio12 (annual precipitation) proved to be the three most influential predictors, with a combined relative importance exceeding 91%. Their optimal ranges were 16.05–29.88 °C for Bio7, 6.53–2513.17 m for altitude, and below approximately 1800 mm for Bio12, indicating a preference for subtropical mountain habitats with moderate seasonal temperature variation and well-drained conditions. (2) The present-day high-suitability zones lie predominantly within three provincial-level regions (Yunnan, Guizhou, and Sichuan) plus parts of southern Gansu and southeastern Fujian, spanning 61.16 × 10^4^ km^2^. (3) Future projections reveal a clear emission-dependent divergence. Under the low-emission pathway (SSP126), suitable habitat fluctuates but partially recovers by the 2090s. Under the most extreme pathway (SSP585), suitable habitat shows persistent contraction, with the high-suitability extent declining to 7.91 × 10^4^ km^2^ by the 2050s, a reduction of 87.07% relative to the present day, while the distributional core shifts more than 200 km northwestward. Across all scenarios, the centroid consistently migrates northwestward, though distances vary from 38.47 km (SSP245) to over 200 km (SSP585). Conservation priority should be given to retention zones in Yunnan, Guizhou, and Sichuan, whereas potential expansion areas in Shaanxi, Henan, and Shandong warrant close surveillance. These findings provide a quantitative basis for cultivation regionalization, wild resource conservation, and climate-adaptation strategies for *B. officinalis*.

## Simple Summary

Climate change is changing where plants can grow. *Buddleja officinalis* Maxim. (*B. officinalis*) is a shrub from southern China. Its flower buds are used in traditional medicine, and its flowers feed pollinators. Wild populations are declining. We collected 346 locations of this plant and combined them with climate, terrain, vegetation, and human-activity data. We used a computer model to map where it can grow today and where it may grow in the future under four possible climate pathways. We found that seasonal temperature differences, elevation, and yearly rainfall explain most of its distribution. Its best-growing areas are currently in Yunnan, Guizhou, and Sichuan. Under the most extreme warming pathway, these areas could shrink by nearly ninety percent by the late twenty-first century, and its range center could move more than two hundred kilometers northwest. Our results help identify places to protect this plant and where to grow it in the future.

## 1. Introduction

The geographic ranges of plant species are being progressively redefined by global climate shifts. Research indicates that between 2000 and 2020, the mean global surface temperature rose by about 1.0 °C relative to pre-industrial baselines [1]. In this context, a substantial body of research has confirmed that climate change is causing the distribution boundaries of plant species to shift towards polar regions or higher elevations, with migration rates particularly significant in mid-to-high latitudes [2,3,4]. It is estimated that if global warming reaches 2 °C, approximately 16% of plant species will face the risk of a >50% reduction in their suitable habitat area, with endemic species in tropical and subtropical regions being most severely threatened [5,6]. For species with important ecological functions and economic value, warming-induced habitat shrinkage and fragmentation may further exacerbate the loss of population genetic diversity, thereby weakening their long-term adaptive capacity [7,8]. Therefore, accurately predicting shifts in suitable habitats under climate change has become a core issue in conservation biology, biogeography, and resource management science [9,10]. Because these impacts are spatially heterogeneous and species-specific, such predictions are particularly urgent for economically and ecologically important plants whose suitable areas may contract, fragment, or shift across administrative boundaries.

Among the various modeling approaches developed for this purpose, MaxEnt has emerged as a premier choice for habitat suitability modeling [11,12]. This model operates on presence-only records and demands minimal sample sizes, yet delivers robust predictive performance even with limited data [13,14]. In particular, MaxEnt can effectively handle complex nonlinear relationships between continuous and categorical environmental variables and can quantitatively evaluate the contribution rates and suitable thresholds of each environmental factor through response curves [15,16,17]. Moreover, its prediction results are output as continuous probabilities, facilitating spatial visualization and zonal statistics within GIS platforms. These advantages have led to the widespread application of MaxEnt in the conservation of rare and endangered plants, early warning of invasive species risks, and planting regionalization of medicinal plants [18,19,20,21,22].

*Buddleja officinalis* Maxim. (*B. officinalis*) is a deciduous shrub belonging to the genus *Buddleja* (Scrophulariaceae) and is an important native plant resource in Southwest and South China [23,24]. Medicinally, the flower buds of *B. officinalis* serve as a traditional Chinese remedy for pyrexia, visual disturbances, and pain relief. Modern pharmacological studies have shown that the active constituents of *B. officinalis*, including flavonoids and phenylethanoid glycosides, exhibit beneficial anti-inflammatory, antioxidant, and neuroprotective effects [25,26,27]. As an excellent nectar plant, *B. officinalis* is characterized by a long flowering period and abundant nectar production, which plays a positive role in maintaining regional pollination networks [28]. However, in recent years, due to habitat destruction and over-collection, its wild resources have faced considerable pressure, underscoring the urgent need for systematic resource assessment and conservation planning.

Although its medicinal and ecological value is well recognized, the ecological context and adaptive capacity of *B. officinalis* remain insufficiently understood. Ecologically, the species is predominantly distributed across subtropical and warm-temperate regions of China, with natural populations concentrated in Yunnan, Guizhou, Sichuan, Guangxi, and adjacent areas [29]. It typically occurs in open habitats such as shrublands, forest margins, roadside slopes, and riverbanks, at elevations ranging from approximately 200 to 2800 m. The species prefers well-drained soils and moderate seasonal temperature variation, and is relatively tolerant of drought and poor soil conditions, which partly explains its wide but patchy distribution across mountainous southwestern China. In the study area, its occurrence is closely associated with the complex topography and monsoon climate of the Yunnan–Guizhou Plateau, where mild and humid conditions prevail without pronounced seasonal drought. Nevertheless, the relative importance of temperature annual range, precipitation, and elevation in constraining its distribution limits has not been quantitatively assessed.

Despite the recognized medicinal and ecological importance of *B. officinalis*, its biogeographic patterns and climate-change responsiveness remain largely unexplored systematically. Previous studies on *B. officinalis* have primarily concentrated on its phytochemistry, pharmacology, and pollination biology, whereas its cultivation techniques and germplasm evaluation remain largely descriptive and regionally limited. By contrast, studies on its geographic distribution, ecological niche, and climate-change responses are notably scarce. Specifically, three key questions remain unanswered. First, the key environmental drivers shaping the habitat of *B. officinalis* remain ambiguous, largely owing to insufficient quantitative assessment of how multiple predictor groups (climate, topography, vegetation, and anthropogenic factors) contribute comparatively. Second, the ecological suitability thresholds for the dominant factors (e.g., temperature annual range, annual precipitation, elevation range) lack quantitative definition, leaving our understanding of its niche breadth at a purely empirical, descriptive level. Third, no systematic predictions have been made regarding the spatial reorganization (retention, loss, and expansion) of *B. officinalis* suitable habitats, nor the centroid shifts of its range under alternative future climate projections. Notably, although MaxEnt has been extensively applied to predict the potential ranges of medicinal plant species [30,31,32,33], no analogous study has yet been conducted for *B. officinalis*. Answering the above questions has important scientific implications for the cultivation regionalization, wild resource conservation, and climate change adaptation strategy development for *B. officinalis*.

To address these questions, this study integrates 24 environmental variables (spanning climatic, topographic, vegetative, and anthropogenic categories) with the latitude and longitude data of 346 occurrence points of *B. officinalis*. Using an ENMeval-optimized MaxEnt approach, we projected the potential distribution of *B. officinalis* across China under both current and future climates across three future periods and four emission scenarios. Specifically, this study was designed with three primary goals: (1) to pinpoint the key environmental determinants of *B. officinalis* distribution, assess their relative contributions, and establish their optimal ranges via response curve analysis; (2) to produce current-climate suitability maps classified into three tiers (high, moderate, and low), and to estimate both the area and national percentage of each tier; and (3) to examine how suitable areas for *B. officinalis* will change in size and spatial configuration (whether expanding, lost, or persisting) across future climate scenarios, and to track how far and in which direction its range centroid will shift. Ultimately, these findings are expected to support the sustainable utilization of *B. officinalis* through improved conservation practices, optimized cultivation zones, and adaptive responses to climate change.

## 2. Materials and Methods

### 2.1. Compilation and Cleaning of B. officinalis Presence Records

**Figure 1:**
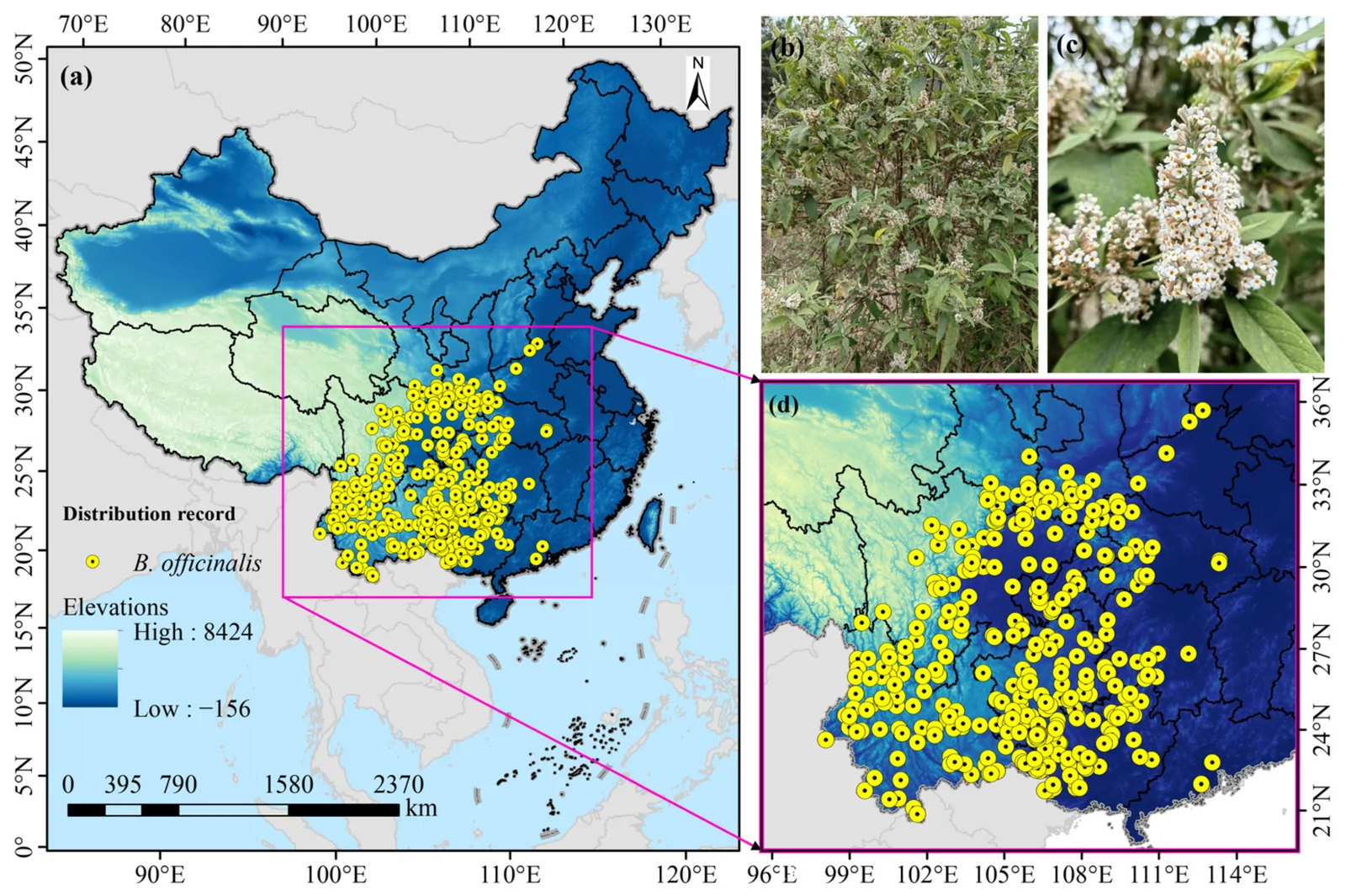
Georeferenced occurrence records of *B. officinalis* (n = 346) within China. (**a**) Spatial distribution of occurrence records overlaid on an elevation gradient; (**b**) field photograph of *B. officinalis* in its natural habitat; (**c**) Close-up of the inflorescence; (**d**) enlarged view of the southwestern China region (approximately 96–114° E, 20–38° N), where the species is most concentrated. Occurrence records were compiled from GBIF, CVH, and CNKI, and spatially filtered to retain only the record nearest the centroid within each 2.5° × 2.5° grid cell.

Publicly available records for *B. officinalis* in China were sourced from publicly accessible literatures and online repositories, specifically the Global Biodiversity Information Facility (GBIF, https://doi.org/10.15468/dL.3s7nbn), the Chinese Virtual Herbarium (CVH, http://www.cvh.ac.cn/), and the China National Knowledge Infrastructure (CNKI, https://www.cnki.net/). Using the online geocoding service “Jingweidu” (http://jingweidu.757dy.com/), we assigned coordinates to occurrence records that provided only village-level toponyms. After applying Excel’s deduplication function to eliminate coordinate-wise duplicates, we obtained 387 unique points (232 GBIF, 137 CVH, 18 CNKI). We reduced the influence of sampling bias and redundant occurrences on model outputs by spatially filtering the presence points with ArcGIS 10.8. To ensure spatial representativeness, we kept only the occurrence record nearest the centroid within each 2.5′ × 2.5′ grid cell. This process resulted in 346 points for subsequent modeling (198 from GBIF, 130 from CVH, and 18 from CNKI) (Figure 1; Table S1). The filtered occurrence records were then compiled into a CSV file conforming to MaxEnt input specifications. The study area was defined as the terrestrial territory of China, and all spatial analyses followed the standard reporting protocol for species distribution models [34]. Base maps were obtained from the Ministry of Natural Resources Standard Map Service (review license No. GS (2023) 2762; http://bzdt.ch.mnr.gov.cn), and the original boundaries were strictly preserved without modification.

### 2.2. Environmental Variable Selection and Preparation

We assembled 24 environmental variables (Table S1) representing climatic, topographic, vegetative, and anthropogenic factors to comprehensively assess their effects on the distribution of *B. officinalis*. The 19 bioclimatic variables and elevation data were obtained from WorldClim 2.1 (https://www.worldclim.org/) at 2.5 arc-minute resolution (approximately 4.5 km at the equator), derived from monthly temperature and precipitation data for the baseline period 1970–2000 [35]. Slope and aspect were derived from elevation in ArcGIS 10.8. NDVI (MOD13A3, 1 km) monthly composites for 2000–2018 were aggregated via maximum-value compositing into a long-term mean layer [36]. The Human Footprint Index (1 km, 2000–2018) was derived by summing eight normalized human pressure layers following Venter et al. [37], with the period represented by the multi-year mean. All layers were resampled to a uniform 2.5 arc-minute grid using bilinear interpolation, clipped to China’s terrestrial boundary, projected to WGS 1984, and co-registered to a common grid. Categorical variables used nearest-neighbor resampling. Future projections used the BCC-CSM2-MR model under four SSP scenarios (SSP126, SSP245, SSP370, SSP585) for three time slices (2050s, 2070s, 2090s), all bias-corrected and downscaled by WorldClim using the delta method, ensuring direct comparability with historical layers. The BCC-CSM2-MR model was selected for its proven performance in reproducing temperature and precipitation patterns across China [38]. Multi-model ensemble approaches, though not adopted here, are recommended in future studies to better capture inter-model uncertainty.

**Figure 2:**
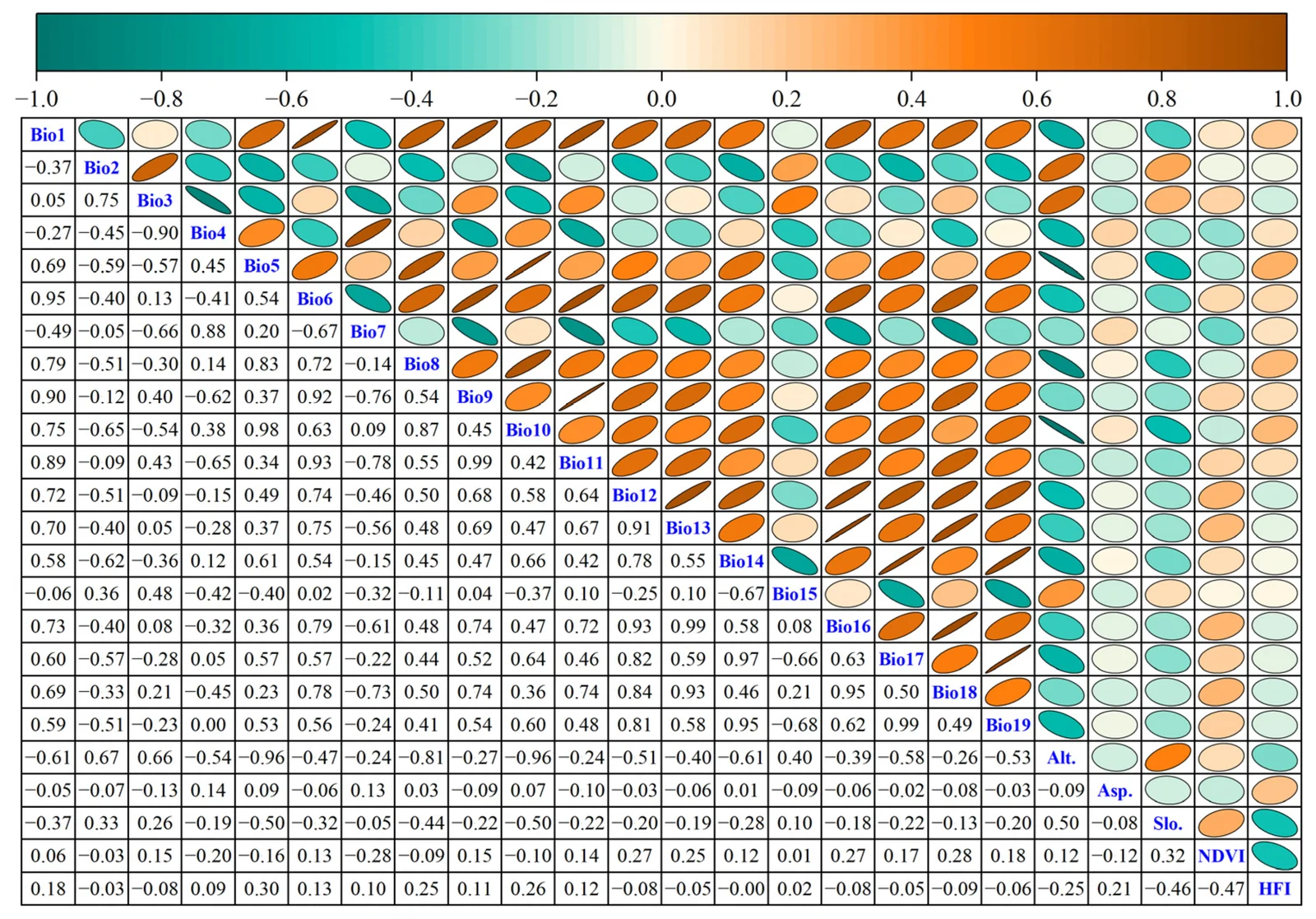
Correlation matrix of the 24 candidate environmental layers (Spearman’s rank). Flatter brown ellipses correspond to stronger positive correlations, whereas flatter cyan ellipses reflect stronger negative correlations. Ele. and Asp. stand for elevation and aspect, respectively. Table S1 lists the full names and units of all bioclimatic variables (Bio1–Bio19).

To prevent predictor collinearity from compromising model performance [39], we adopted a strict variable selection strategy. Step 1: We ran the 346 occurrence points and 24 layers in MaxEnt (v3.4.4) software (https://biodiversityinformatics.amnh.org/open_source/maxent/, accessed 29 January 2026) using 25% test data, a regularization multiplier of 1, and 10 replicates, with all other parameters at default values. The percent contribution of each variable was obtained based on regularized gain (Table S1). Step 2: After extracting environmental data for each of the 346 occurrence points with ArcGIS 10.8, we conducted a Spearman correlation analysis in Origin 2026 (Figure 2). The 0.7 threshold is widely used to balance redundancy removal and information retention [40]. To further verify that the retained predictors were free from multicollinearity, we calculated the Variance Inflation Factor (VIF) for the eight variables selected by the Spearman correlation procedure. All VIF values were below 10, confirming that the retained variables were sufficiently independent for model construction. Step 3: Following the above procedure, eight representative variables (Bio7, altitude, Bio12, HFI, NDVI, slope, aspect, and Bio15, covering all four environmental categories) were retained from the 24 initial variables (Table S1), with all pairwise correlation coefficients among them less than 0.7. The final model was built using only these eight variables after the correlation-based selection.

It should be noted that future projections were driven solely by changes in climatic variables, whereas NDVI and the Human Footprint Index were held constant at their current values. This assumption was made because spatially explicit future projections of NDVI and HFI under SSP scenarios are not yet available at a comparable resolution. Consequently, future projections reflect the isolated effect of climate change on habitat suitability.

### 2.3. MaxEnt Model Calibration and Projection

#### 2.3.1. Optimizing Model Settings with ENMeval

To prevent biased outputs from uncalibrated models [41], we used the R package ENMeval 2.0 [42] to optimize MaxEnt. We tuned the regularization multiplier (RM; 0.5–4.0, step 0.5) and evaluated nine feature class (FC) combinations (H, HPT, L, LQ, LQH, LQHP, LQHPT, QHP and QHPT) built from five feature types: linear (L), quadratic (Q), product (P), threshold (T), and hinge (H) [43]. We selected the best-fitting model using delta AICc and OR10 [11], ensuring robust predictions of *B. officinalis* distribution under future climates. This RM range and step size follow standard ENMeval practice, allowing gradual complexity reduction while avoiding excessive computation [42]. Delta AICc and OR10, respectively, measure goodness-of-fit and overfitting risk, providing complementary criteria for model selection [44].

#### 2.3.2. Final MaxEnt Model Configuration

Using the optimized parameter combination, we projected the potential distribution of *B. officinalis* with the final MaxEnt model. From the 346 validated occurrence records, 75% (260 points) were randomly assigned for training and 25% (86 points) for testing. This split ratio is widely used in presence-only modeling to balance training robustness and independent evaluation [13]. Model stability was enhanced through 10-fold repeated cross-validation, and 10,000 background points were used to characterize environmental heterogeneity, a number sufficient to capture background variation without excessive computation [12]. To balance complexity with generalization, the regularization multiplier was fixed at 0.5 and feature class restricted to linear and quadratic (LQ) forms, as determined by preliminary optimization. These simpler features reduce overfitting while preserving ecologically interpretable response shapes [42,45]. The final outputs, in logistic probability format, were exported as ASC rasters for subsequent spatial analyses.

#### 2.3.3. Model Performance Assessments

We evaluated the final MaxEnt model using three metrics: the area under the receiver operating characteristic curve (AUC), true skill statistic (TSS), and Cohen’s Kappa coefficient. Using multiple metrics is recommended because each captures a different aspect of model performance: AUC measures overall discrimination, TSS accounts for sensitivity and specificity, and Kappa assesses agreement beyond chance [46,47]. TSS and Kappa were calculated from the same presence-background predictions using the 10th percentile training presence threshold (0.0999) for binarization. TSS = sensitivity + specificity−1 and is prevalence-independent; Kappa = (Po − Pe)/(1 − Pe), with prevalence approximated by the proportion of the study area predicted as suitable, following Allouche et al. [46] for presence-only data. For AUC (range 0–1), scores ≥ 0.7 indicate acceptable predictive ability, with 0.7–0.8 considered fair, 0.8–0.9 good, and ≥0.9 outstanding [48]. TSS values > 0.4 indicate useful predictions, with >0.8 considered excellent [46]. Kappa agreement was classified as poor (<0.4), good (0.4–0.75), or excellent (>0.75) [47]. These threshold criteria follow established conventions in species distribution modeling [46,47,48].

### 2.4. Classification of Species Suitable Areas

To improve model predictive capability, the threshold for delineating suitable versus unsuitable areas was defined as the mean 10th percentile training presence threshold computed from 10 MaxEnt replicate runs [39,49]. We also calculated the Maximum Test Sensitivity Plus Specificity (MTSPS) threshold (0.2389), compared with the 10th percentile training presence threshold (0.0999). We selected the latter as the primary criterion because our occurrence records likely contain spatial sampling bias. Under such conditions, the 10th percentile threshold is more conservative and reduces overprediction [39,50], whereas MTSPS assumes unbiased test data and may overestimate suitable areas. To date, no study has shown MTSPS to outperform the 10th percentile threshold under biased sampling.

The key advantage of this threshold is that it robustly balances model goodness-of-fit to known occurrence points (avoiding overfitting) with generalization ability to unknown habitats. Specifically, it systematically allows a small number of known points located at distribution margins or extreme microhabitats to be classified as non-suitable, thereby effectively reducing overprediction caused by field sampling noise or localized microhabitat extremes. This approach generates suitable area maps that are ecologically more credible and more stable for conservation planning. This method has been validated by methodological studies [50] and subsequently adopted by multiple applied studies [51,52,53]. Using this threshold, we classified the study area into suitable (≥cutoff) and non-suitable (<cutoff) zones, and visualized the resulting binary suitability map. We then transferred the MaxEnt output to ArcGIS 10.8 and applied the Natural Breaks (Jenks) classification to divide the suitable areas into multiple suitability levels. Finally, we categorized the suitability probability into four tiers: non-suitable (0–0.0999), low (0.0999–0.30), medium (0.30–0.50), and high (0.50–1.0).

### 2.5. Spatial Dynamics of Suitable Areas

Based on temporal changes in suitability, we identified three categories of range dynamics: areas that remain suitable across both current and future periods (retention), those shifting from suitable to unsuitable (loss), and those becoming newly suitable (expansion) [54]. We imported the suitability files for both current and future periods into ArcGIS 10.8, then applied a 0.0999 probability cutoff to convert them into binary vector polygons for suitable (≥threshold) and non-suitable zones. The Intersect tool was used to overlay future and current suitable area vector maps, and retention, loss, and expansion areas were identified based on the overlay attribute combinations. The results were then converted back to raster format. The area and proportion of each type were quantitatively calculated, and the retention, contraction, and expansion trends of *B. officinalis* suitable areas under different SSP scenarios (SSP126 to SSP585) and time periods (2050s, 2070s, 2090s) were analyzed.

### 2.6. Centroid Migration of Suitable Areas

Centroid shifts serve as a useful indicator of how species distributions move spatially in response to climate change. To evaluate how *B. officinalis* habitats respond spatially to climate change, we considered a current baseline (1970–2000) and three future windows (2050s, 2070s, 2090s) under four SSP scenarios (SSP126, SSP245, SSP370, SSP585). We followed a three-step procedure for centroid analysis. First, the average MaxEnt prediction rasters were transferred to ArcGIS 10.8 and reclassified into binary format using the 0.0999 threshold. Second, centroids of suitable areas for each scenario and period were derived using ArcGIS geometry tools, and their coordinates were recorded [54]. Third, centroid displacement distances were calculated via Vincenty formula to track spatiotemporal changes in *B. officinalis* habitats under varying climate scenarios [55].

## 3. Results

### 3.1. Model Tuning and Performance Evaluation

**Figure 3:**
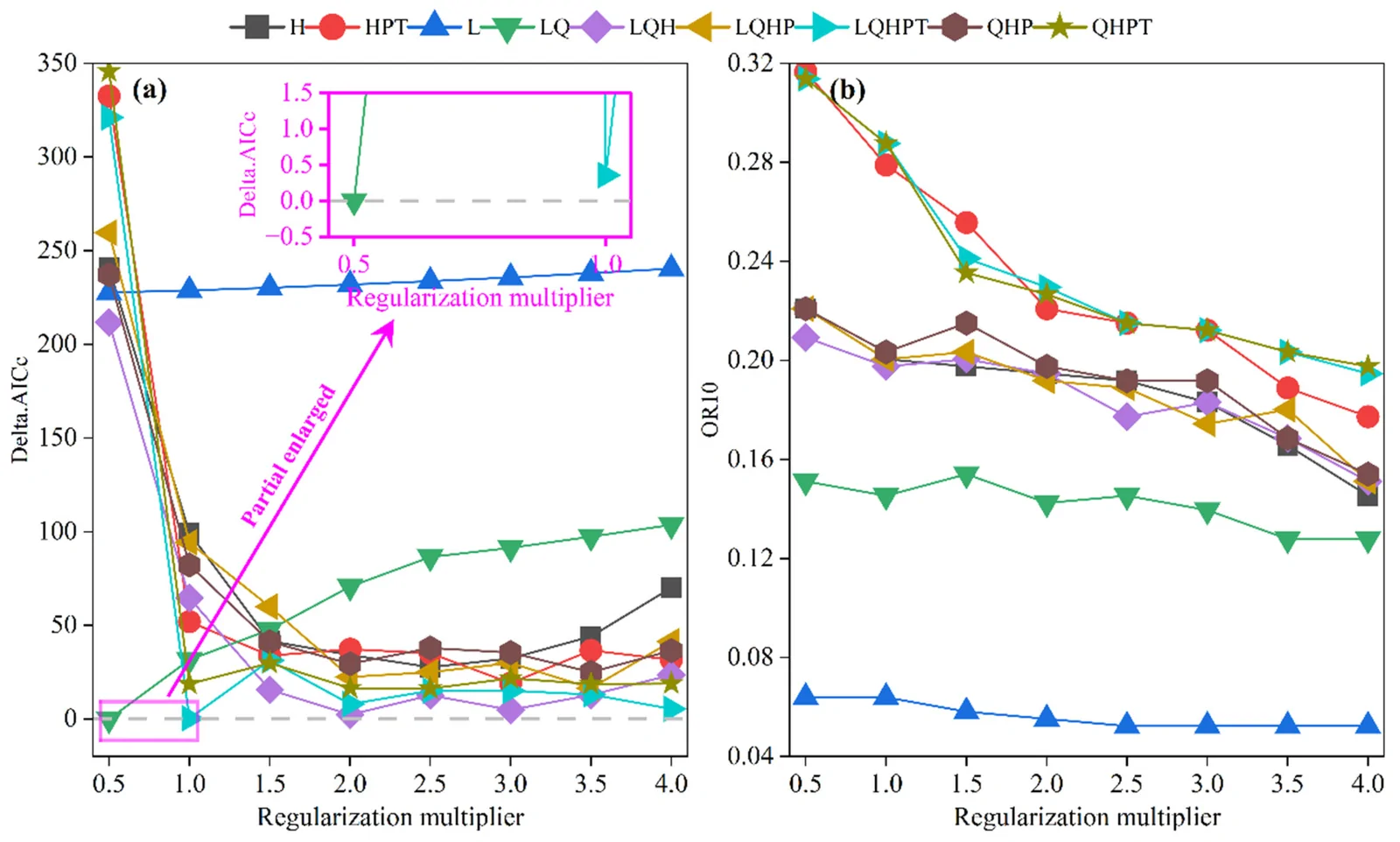
ENMeval-derived evaluation metrics for *B. officinalis* across different regularization multipliers (RM; 0.5–4.0) and feature class combinations. (**a**) Delta AICc values; lower values indicate better model fit relative to complexity, with values < 2 considered equivalent to the best model. (**b**) OR10 values (the 10th percentile of omission rates); lower values indicate lower overfitting risk. Feature classes: L = linear, Q = quadratic, H = hinge, P = product, T = threshold.

To guide our parameter selection, we calculated Delta.AICc and OR10 for each model configuration. The default settings (RM = 1, FC = LQHP) yielded a Delta.AICc of 94.34 (Figure 3a). According to Anderson and Gonzalez [44], a Delta.AICc above 2 signals that the default MaxEnt configuration is inadequate and needs to be re-tuned to enhance model accuracy. ENMeval calibration of the 346 occurrence points and eight environmental variables yielded two Delta.AICc < 2 configurations: RM = 0.5/FC = LQ (Delta.AICc = 0) and RM = 1.0/FC = LQHPT (Delta.AICc = 0.3588). OR10 values were 0.1511 and 0.2877, respectively, with the latter 92.30% higher, indicating better conservatism for the RM = 0.5/FC = LQ model (Figure 3b). The RM = 0.5 with LQ features was selected as the optimal MaxEnt configuration under the parsimony criterion [45]. This set yielded Delta.AICc = 0 (theoretical optimum) and balanced accuracy with model simplicity.

**Figure 4:**
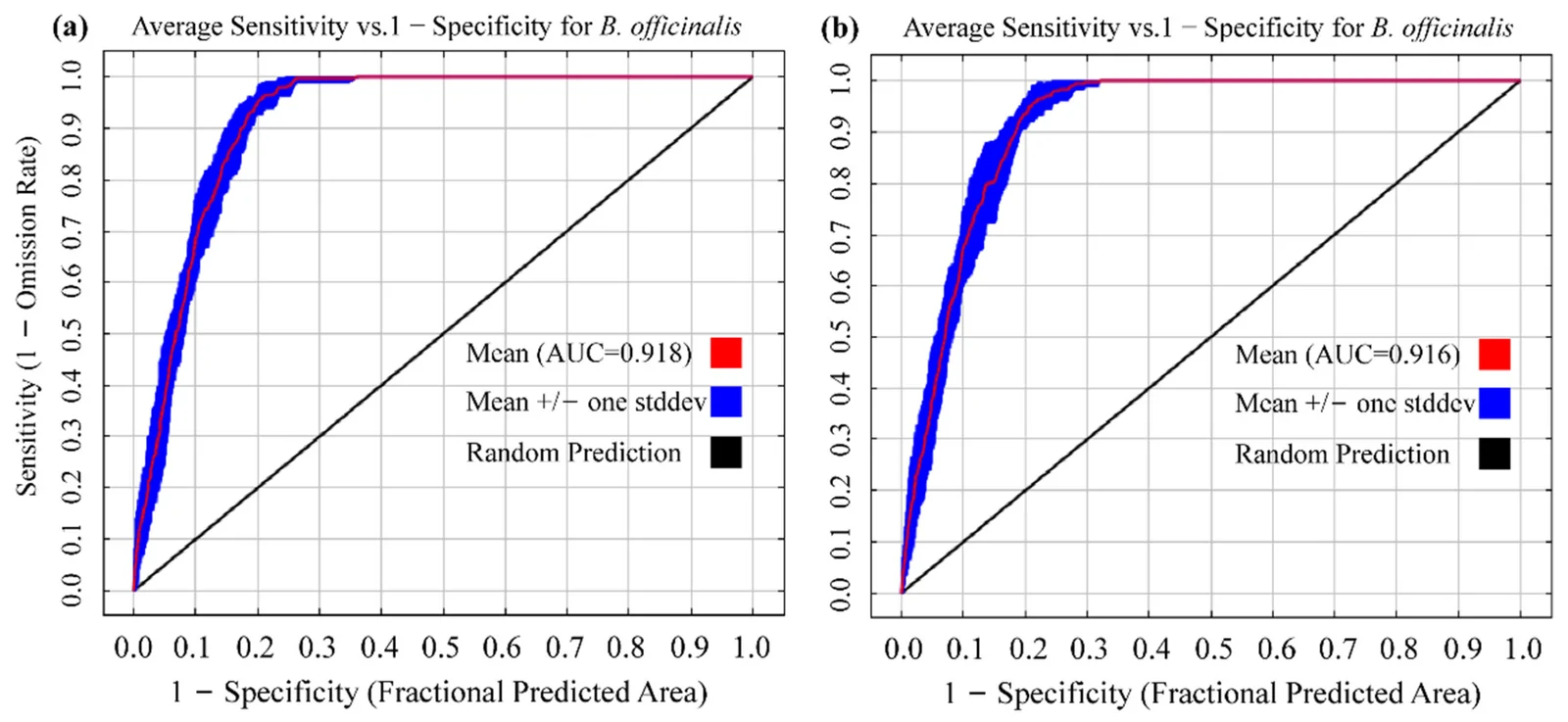
Receiver operating characteristic (ROC) curves and area under the curve (AUC) scores for *B. officinalis* MaxEnt models. (**a**) Default settings (RM = 1, FC = LQHP), with a mean AUC of 0.918. (**b**) Optimized settings (RM = 0.5, FC = LQ), with a mean AUC of 0.916.

We validated the optimized MaxEnt configuration against the default setup using 10 replicate cross-validations. The default parameters (RM = 1, FC = LQHP) yielded an AUC of 0.918 (Figure 4a), while the optimized settings (RM = 0.5, FC = LQ) produced 0.916 (Figure 4b). Both values exceeded the 0.9 threshold for outstanding performance. The optimized model sacrificed a negligible 0.22% in AUC but gained considerably in generalizability, as stronger regularization and simpler features effectively reduced the overfitting observed in the default run. Under standard evaluation metrics, the optimized model recorded a TSS of 0.769 ± 0.008 and a Kappa of 0.417 ± 0.017.

### 3.2. Key Environmental Drivers of B. officinalis Distribution

**Figure 5:**
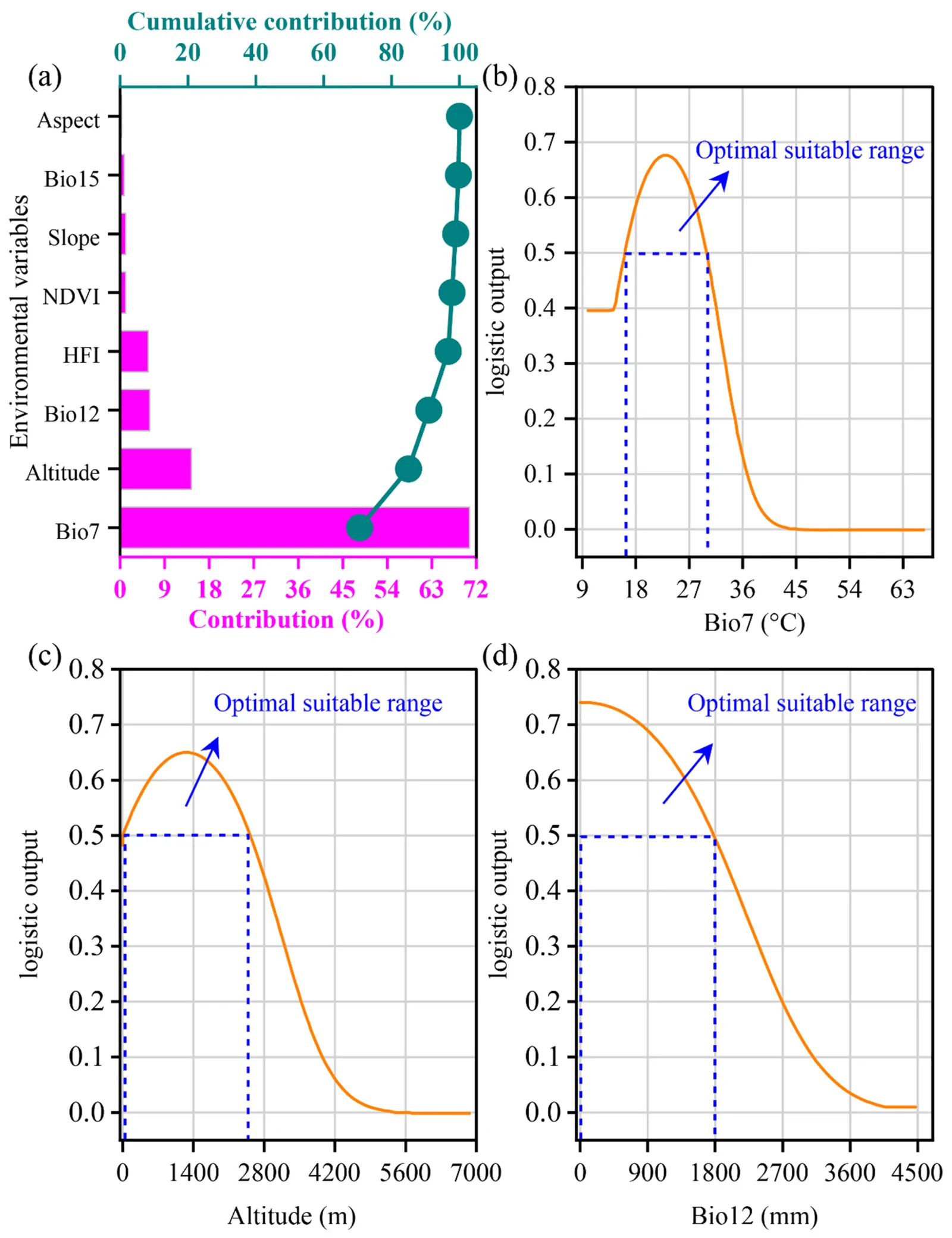
Contribution analysis (**a**) and response curves (**b**–**d**) for *B. officinalis* MaxEnt modeling. The orange lines represent mean suitability across 10 replicate runs. Blue dashed lines mark the optimal ranges for Bio7 (**b**), altitude (**c**), and Bio12 (**d**).

The optimized MaxEnt model allowed us to order the eight environmental variables by their relative influence on *B. officinalis* distribution. The five most influential variables were temperature annual range (Bio7, 70.6%), altitude (14.4%), annual precipitation (Bio12, 6%), HFI (5.7%), and NDVI (1.1%) (Figure 5a). Permutation importance yielded a consistent ranking of the top three variables—Bio7 (75.8%), altitude (16.7%), and Bio12 (2.1%)—confirming that these three factors are the dominant drivers regardless of the importance metric used. The eight environmental factors were classified into four categories: climate, topography, human activity, and vegetation, with corresponding cumulative contribution rates of 77.4%, 15.8%, 5.7%, and 1.1%, respectively. Following the 85% cumulative contribution criterion [56,57], we identified temperature annual range (Bio7), altitude, and annual precipitation (Bio12) as the three dominant factors. These variables jointly accounted for 91% of the total contribution, underscoring their overriding role in determining *B. officinalis* distribution in China.

The occurrence probability of *B. officinalis* responded to Bio7, altitude, and Bio12 in a unimodal fashion, yet each factor exhibited a distinct optimal range (Figure 5b–d). Specifically, for annual temperature range (Bio7), the presence probability of *B. officinalis* remained stable at approximately 0.40 between 9.96 °C and 14.22 °C. It then increased rapidly from 14.22 °C to 23.32 °C, reaching a maximum value of 0.68. Subsequently, it decreased continuously with increasing temperature, dropping to 0 at 42.59 °C and remaining unchanged thereafter (Figure 5b). For altitude, the presence probability fluctuated and increased from 0.48 to a maximum value of 0.65 within the range of 0 to 1283.50 m. It then decreased continuously with increasing altitude, dropping to near 0 at 4799.10 m and remaining at 0 thereafter (Figure 5c). For annual precipitation (Bio12), the presence probability remained stable at its highest value of 0.74 within the range of 0 to 61.90 mm. It then decreased continuously with increasing precipitation, reaching approximately 0.70 at 793.31 mm. The decreasing trend accelerated when precipitation exceeded 910.76 mm, and the probability dropped to 0.01 at 4003.38 mm, after which it stabilized (Figure 5d). According to the criterion of Liu et al. [58], which defines the most suitable habitat as having a presence probability ≥ 0.5, the optimal suitability intervals for *B. officinalis* are: Bio7 between 16.05 °C and 29.88 °C, altitude between 6.53 m and 2513.17 m, and Bio12 between 0 and 1783.76 mm (Figure 5b–d).

### 3.3. Present Habitat Suitability for B. officinalis Across China

**Figure 6:**
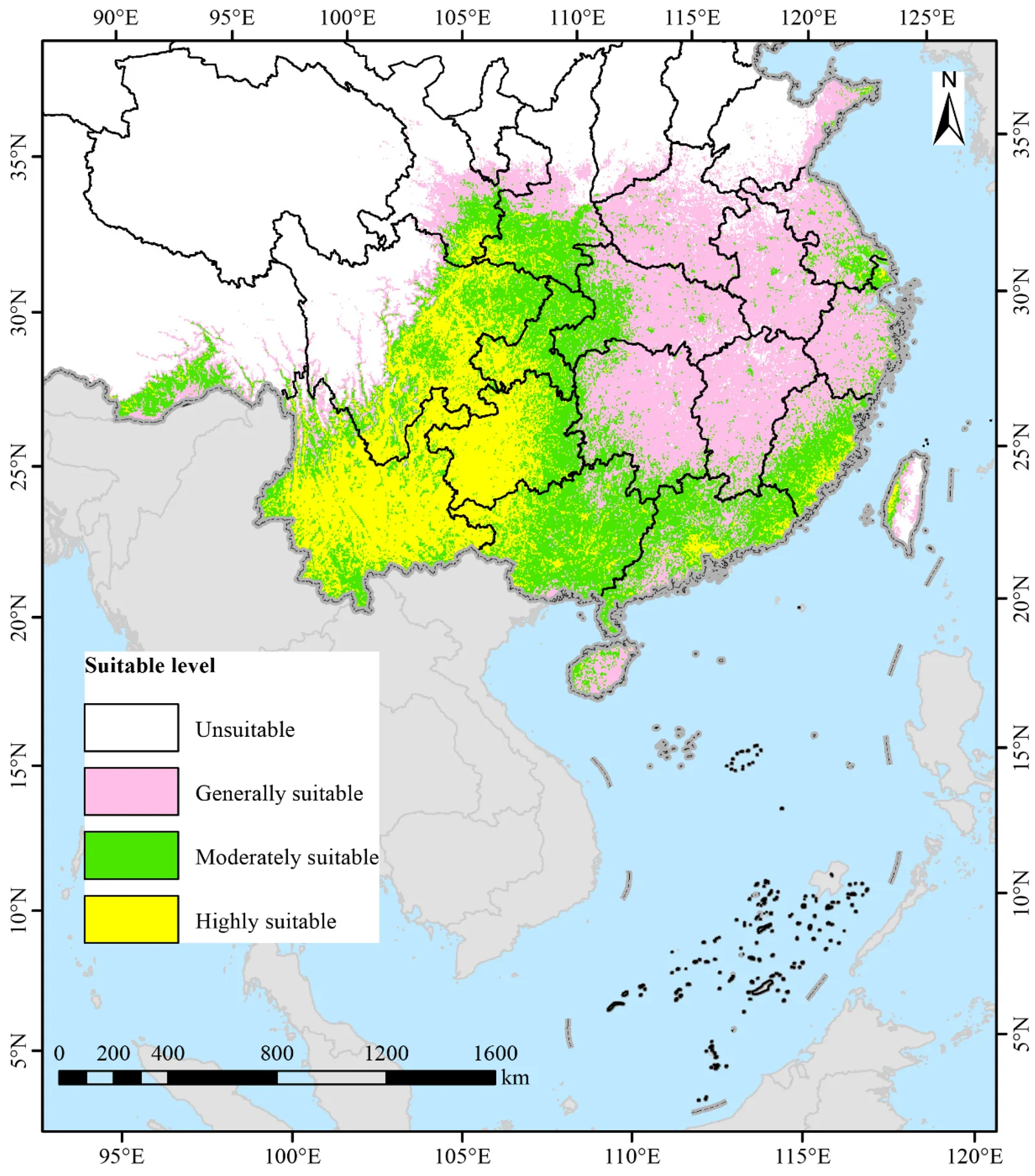
Predicted current suitable habitats for *B. officinalis* across China, classified into four tiers using the 10th percentile training presence threshold and Natural Breaks (Jenks) classification: unsuitable (white), low suitability (pink), moderate suitability (green), and high suitability (yellow).

Current climate supports about 263.61 × 10^4^ km^2^ of potential *B. officinalis* habitat in China (27.46% of the country). This estimate represents the climatically permissible envelope rather than the realized range, which is concentrated in southwestern and southern China (Figure 1). The discrepancy likely reflects overprediction inherent to presence-background MaxEnt when the species is not in climatic equilibrium, and, to a lesser extent, the coarse 2.5 arc-minute resolution. The high-suitability core (6.37% of China) is therefore a more conservative, management-relevant estimate. The three suitability classes (high, medium, and low) were arranged in a nested pattern, with suitability gradually declining from core to periphery (Figure 6). More specifically: (1) high-suitability areas (61.16 × 10^4^ km^2^, 6.37% of China) are mainly distributed across the southwestern and southern provinces, including Yunnan, Guizhou, Sichuan, and Guangdong, with additional patches in Gansu, Shaanxi, Guangxi, Fujian, and Taiwan. (2) medium-suitability habitats covered 87.06 × 10^4^ km^2^ (9.07% of China), encircling the high-suitability zones across southern and central-eastern China. Key provinces include Guangxi, Guangdong, Fujian, Guizhou, Chongqing, Hubei, Shaanxi, Jiangsu, Taiwan, and Tibet. (3) low-suitability habitat was the most extensive category, occupying 115.40 × 10^4^ km^2^ (12.02%) and occurring mainly along the fringes of medium-suitability areas and throughout eastern provinces, including Hunan, Jiangxi, Anhui, Zhejiang, Henan, eastern Hainan, eastern Hubei, northern Jiangsu, and eastern Shandong.

### 3.4. Climate-Driven Changes in Future Suitable Areas for B. officinalis

**Figure 7:**
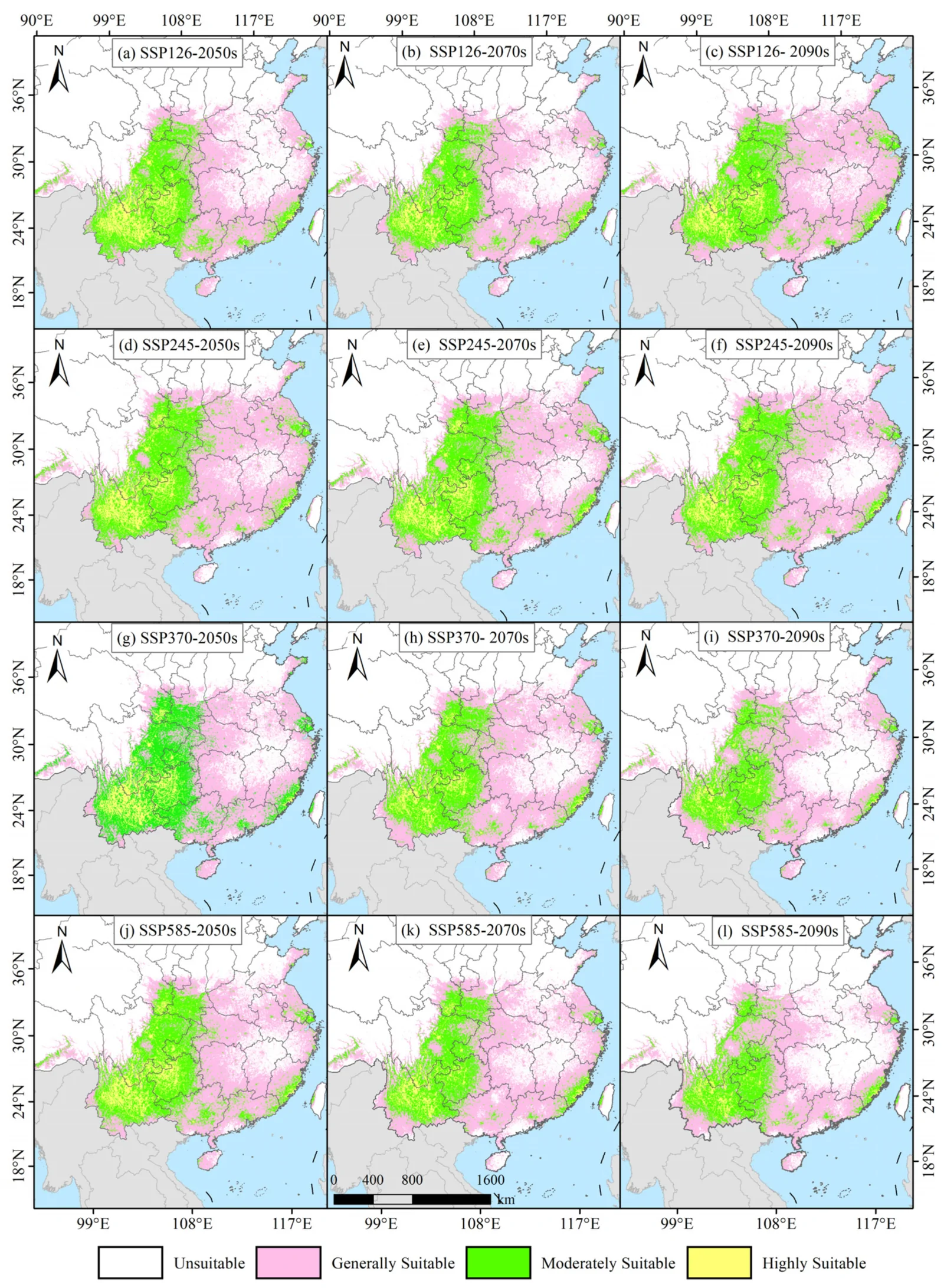
Predicted future suitable habitats for *B. officinalis* under the SSP126-SSP585 scenarios in the 2050s−2090s, classified into four tiers using the 10th percentile training presence threshold and Natural Breaks (Jenks) classification: unsuitable (white), low suitability (pink), moderate suitability (green), and high suitability (yellow). (**a**) SSP126-2050s, (**b**) SSP126-2070s, (**c**) SSP126-2090s, (**d**) SSP245-2050s, (**e**) SSP245-2070s, (**f**) SSP245-2090s, (**g**) SSP370-2050s, (**h**) SSP370-2070s, (**i**) SSP370-2090s, (**j**) SSP585-2050s, (**k**) SSP585-2070s, and (**l**) SSP585-2090s.

Under the SSP126 climate scenario, projections indicate that the total potentially suitable habitat for *B. officinalis* will reach 207.41 × 10^4^ km^2^ in the 2050s, equivalent to 21.61% of China’s terrestrial territory (Table 1). This total comprises 118.82 × 10^4^ km^2^ of low-suitability, 65.52 × 10^4^ km^2^ of medium-suitability, and 23.07 × 10^4^ km^2^ of high-suitability areas. Geographically, the high-suitability zones are predominantly located in Yunnan, western Guizhou, southern Sichuan, southwestern Chongqing, and southeastern Fujian (Figure 7a). By the 2070s, the overall suitable area contracts marginally to 205.53 × 10^4^ km^2^ (21.41% of the national total), with suitability classes subdivided into 129.86 × 10^4^ km^2^, 60.21 × 10^4^ km^2^, and 15.46 × 10^4^ km^2^, respectively. The spatial configuration of high-suitability regions during this period remains broadly similar to that observed in the 2050s (Figure 7b). Moving to the 2090s, the aggregate suitable extent expands noticeably to 242.55 × 10^4^ km^2^ (25.27% of the country’s area), where low-, medium-, and high-grade zones account for 155.57 × 10^4^ km^2^, 68.24 × 10^4^ km^2^, and 18.74 × 10^4^ km^2^, respectively. Despite these quantitative fluctuations, the principal distribution of high-suitability habitats persists in the aforementioned regions throughout the entire projection period (Figure 7c).

Under the SSP245 climate trajectory, the predicted suitable area for *B. officinalis* is 247.88 × 10^4^ km^2^ in the 2050s, amounting to 25.82% of China’s territory. Low-, medium-, and high-suitability zones, respectively, contribute 151.60 × 10^4^, 74.38 × 10^4^, and 21.90 × 10^4^ km^2^ to this total. High-suitability habitats are chiefly located in Yunnan, west Guizhou, east Sichuan, southwest Chongqing, south Gansu, and southeast Fujian (Figure 7d). In the 2070s, the total suitable area falls modestly to 242.81 × 10^4^ km^2^ (25.29%), split into 146.42 × 10^4^, 75.09 × 10^4^, and 21.29 × 10^4^ km^2^ for low, medium, and high classes, while the spatial configuration of the highest-grade zones’ parallels that of the 2050s (Figure 7e). By the 2090s, the total further declines to 233.19 × 10^4^ km^2^ (24.29%), with the three suitability levels measuring 143.27 × 10^4^, 70.39 × 10^4^, and 19.54 × 10^4^ km^2^, respectively. Notably, despite the decreasing trend in overall area, the core distribution of high-suitability regions stays consistently anchored in the same geographic areas across all three periods (Figure 7f).

According to the SSP370 climate pathway, the predicted suitable range for *B. officinalis* in the 2050s is 227.11 × 10^4^ km^2^, accounting for 23.66% of China’s land surface. Broken down by suitability level, low-grade zones contribute 132.66 × 10^4^ km^2^, medium-grade contribute 71.56 × 10^4^ km^2^, and high-grade contribute 22.89 × 10^4^ km^2^. These high-suitability areas are mostly found in Yunnan, western Guizhou, eastern Sichuan, southwestern Chongqing, southern Gansu, and southeastern Fujian (Figure 7g). Moving to the 2070s, the total suitable territory drops slightly to 224.55 × 10^4^ km^2^ (23.39%), with the three categories totaling 145.88 × 10^4^, 64.92 × 10^4^, and 13.76 × 10^4^ km^2^, respectively, while the distribution of the upper-tier habitats remains essentially unchanged from the earlier decade (Figure 7h). By the 2090s, the overall suitable area declines more markedly to 190.65 × 10^4^ km^2^ (19.86%), comprising 130.69 × 10^4^ km^2^ of low, 52.02 × 10^4^ km^2^ of medium, and 7.94 × 10^4^ km^2^ of high suitability. Across this last period, the high-suitability zones are still chiefly confined to Yunnan, western Guizhou, eastern Sichuan, southwestern Chongqing, and southeastern Fujian, with southern Gansu dropping out of the core distribution (Figure 7i).

Under the SSP585 climate trajectory, the total suitable territory for *B. officinalis* in the 2050s is estimated at 232.32 × 10^4^ km^2^, which translates to 24.20% of China’s terrestrial area. Broken down by suitability level, low, medium, and high classes, respectively, account for 138.40 × 10^4^, 72.27 × 10^4^, and 21.65 × 10^4^ km^2^. The high-grade zones are largely situated in Yunnan, west Guizhou, east Sichuan, southwest Chongqing, south Gansu, and southeast Fujian (Figure 7j). In the 2070s, the overall suitable area diminishes to 211.13 × 10^4^ km^2^ (i.e., 21.99%), with the three tiers summing to 138.14 × 10^4^, 60.35 × 10^4^, and 12.64 × 10^4^ km^2^, respectively, while the spatial arrangement of the highest-quality habitats remains effectively consistent with that seen in the earlier period (Figure 7k). Moving into the 2090s, the total suitable range contracts further to 177.87 × 10^4^ km^2^, representing 18.53% of the nation, compartmentalized into 119.81 × 10^4^ km^2^ low, 50.15 × 10^4^ km^2^ medium, and 7.91 × 10^4^ km^2^ high suitability. During this latest period, the high-suitability areas shrink geographically, retaining a presence solely in Yunnan, western Guizhou, southern Sichuan, southwestern Chongqing, and southeastern Fujian, whereas the previously occupied parts of southern Gansu are no longer classified as highly suitable (Figure 7l).

### 3.5. Spatiotemporal Variations in Suitable Zones for B. officinalis Under Alternative Climate Scenarios

**Figure 8:**
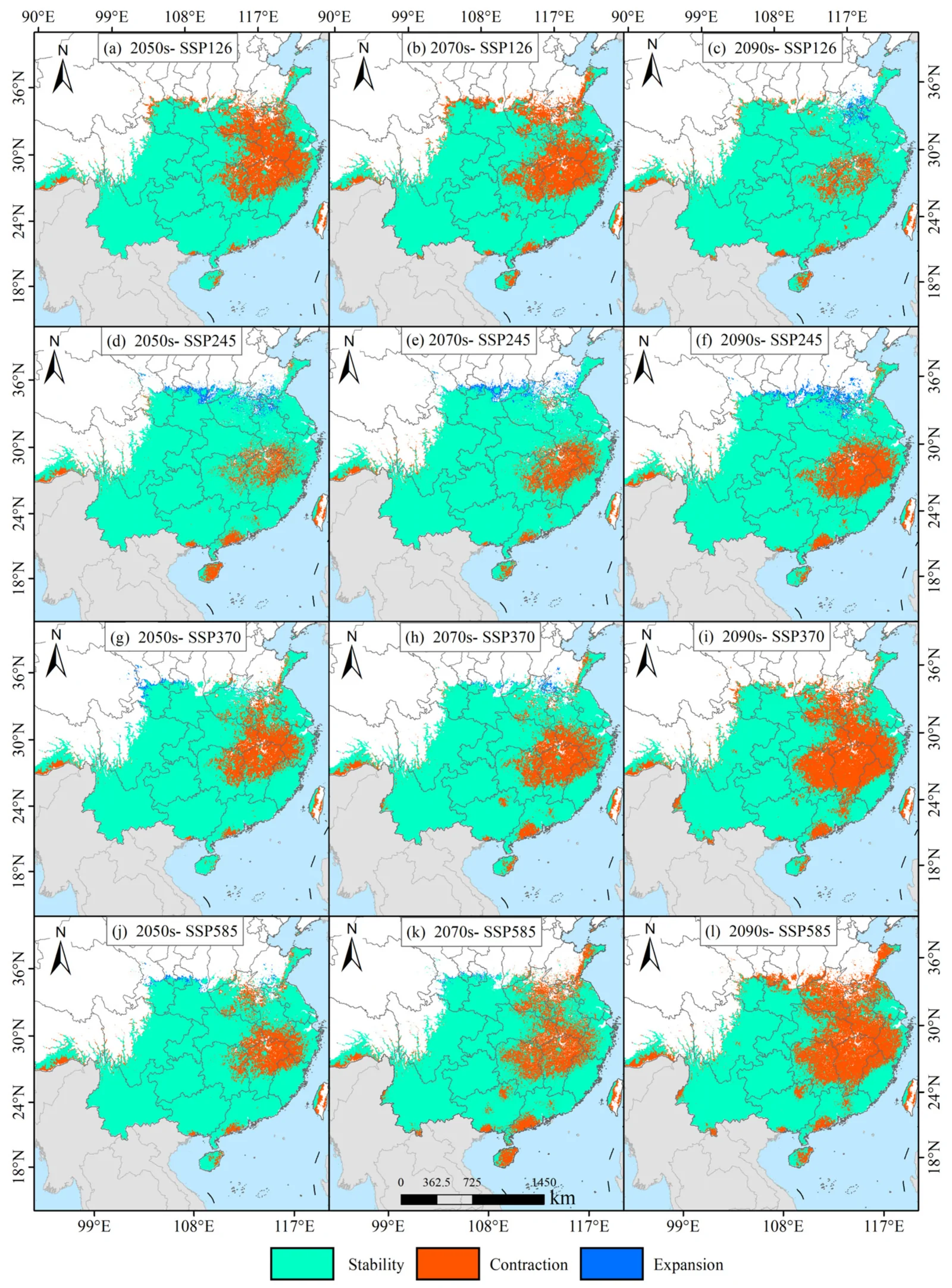
Spatiotemporal dynamics of suitable habitats for *B. officinalis* under four Shared Socioeconomic Pathways (SSP126, SSP245, SSP370, SSP585) across three time slices (2050s, 2070s, 2090s). Panels (**a**–**c**): SSP126; (**d**–**f**): SSP245; (**g**–**i**): SSP370; (**j**–**l**): SSP585. Colors indicate retention (teal), contraction (red), and expansion (blue) relative to the current suitable area. Retention refers to areas suitable in both current and future periods; contraction refers to areas suitable currently but not in the future; expansion refers to areas unsuitable currently but suitable in the future.

According to the SSP126 pathway, the portion of *B. officinalis* suitable habitat that remains stable in the 2050s covers 205.68 × 10^4^ km^2^ (i.e., 77.73% retention), whereas the zones that disappear constitute 58.65 × 10^4^ km^2^ (22.16% loss), and the newly emerging areas represent just 0.28 × 10^4^ km^2^ (0.11% gain). By the 2070s, the stable area experiences a slight reduction to 204.88 × 10^4^ km^2^ (77.45%), the lost area expands to 59.41 × 10^4^ km^2^ (22.46%), and the newly suitable land shrinks further to 0.24 × 10^4^ km^2^ (0.09%). Turning to the 2090s, the retained habitat recovers dramatically to 240.91 × 10^4^ km^2^ (90.12%), the depleted area contracts sharply to 23.42 × 10^4^ km^2^ (8.76%), and the expansion area rises to 2.98 × 10^4^ km^2^ (1.11%) (Table 2). Viewed over the whole study period, this scenario reveals a clear trajectory: stable habitat decreases minimally before undergoing a substantial upturn, lost habitat follows an initial increase and then a steep drop-off, while newly suitable areas first edge downward and then regain momentum (Figure 8a–c).

According to the SSP245 climate trajectory, the enduring suitable zones for *B. officinalis* in the 2050s amount to 241.16 × 10^4^ km^2^, equivalent to an 89.00% retention. Meanwhile, the territories lost are estimated at 22.70 × 10^4^ km^2^ (8.38% loss), and the newly suitable areas add up to 7.10 × 10^4^ km^2^ (2.62% expansion). Moving into the 2070s, the persisting habitat diminishes to 236.43 × 10^4^ km^2^ (87.40%), accompanied by a rise in disappearance to 27.54 × 10^4^ km^2^ (10.18%) and a small reduction in newly gained land to 6.55 × 10^4^ km^2^ (2.42%). By the 2090s, the stable portion falls further to 225.61 × 10^4^ km^2^ (82.94%), the lost zones expand to 38.70 × 10^4^ km^2^ (14.23%), and the expansion area edges back up to 7.69 × 10^4^ km^2^ (2.83%) (Table 2). Overall, this pathway exhibits a monotonic decrease in retained habitat, a progressive rise in lost territory, and a pattern of initial contraction followed by slight recovery in newly suitable zones (Figure 8d–f).

According to the SSP370 climate pathway, the stable suitable land for *B. officinalis* in the 2050s covers 224.38 × 10^4^ km^2^, which translates to a retention of 84.16%. Conversely, the areas that drop out of suitability total 39.98 × 10^4^ km^2^ (14.99% loss), and the newly colonizable regions amount to a mere 2.27 × 10^4^ km^2^ (0.85% expansion). In the 2070s, the persisting habitat edges downward to 222.56 × 10^4^ km^2^ (83.51%), while the lost areas climb to 41.76 × 10^4^ km^2^ (15.67%) and the expansion zones dip to 2.18 × 10^4^ km^2^ (0.82%). By the 2090s, the retained habitat suffers a steep reduction to 189.60 × 10^4^ km^2^ (71.66%), the depleted zones soar to 74.72 × 10^4^ km^2^ (28.24%), and the newly suitable areas crash to an insignificant 0.27 × 10^4^ km^2^ (0.10%) (Table 2). In essence, this climate trajectory reveals a persistent downward slide in stable habitat, a runaway acceleration in habitat loss, and an expansion component that shrinks toward extinction (Figure 8g–i).

Under the SSP585 projection, the stable habitat of *B. officinalis* persisting in the 2050s is quantified at 230.38 × 10^4^ km^2^, corresponding to a retention proportion of 86.50%. During this same period, the areas eliminated from suitability total 33.94 × 10^4^ km^2^ (12.74% loss), while the newly suitable land adds a marginal 2.01 × 10^4^ km^2^ (0.75% gain). Progressing to the 2070s, the persisting fraction declines to 209.54 × 10^4^ km^2^ (79.00%), accompanied by a marked increase in disappeared zones to 54.23 × 10^4^ km^2^ (20.45%) and a contraction of expansion zones to 1.48 × 10^4^ km^2^ (0.56%). By the 2090s, the retained habitat undergoes a further drastic reduction to 176.89 × 10^4^ km^2^ (66.97%), the lost areas escalate substantially to 86.97 × 10^4^ km^2^ (32.93%), and the newly colonized zones dwindle to a negligible 0.29 × 10^4^ km^2^ (0.11%) (Table 2). Taken collectively, this high-emission trajectory is defined by a sustained and substantial erosion of persisting habitat, a relentless upward creep of lost territories, and a continuous attenuation of expansion areas (Figure 8j–l).

### 3.6. Shifts in Centroid Locations of B. officinalis Suitable Habitats Under Climate Projections

**Figure 9:**
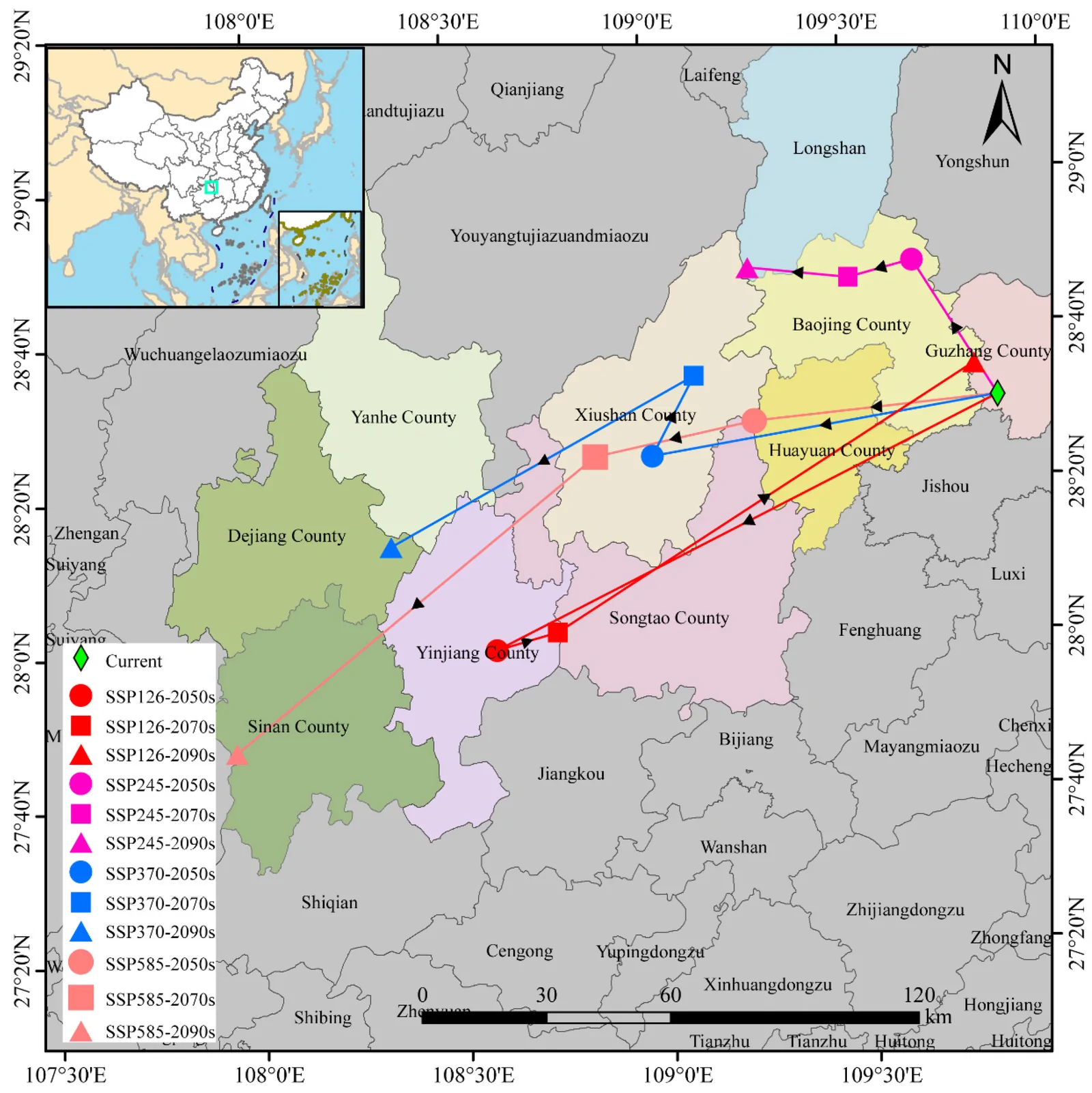
Comparative centroid movement patterns of *B. officinalis* under alternative climate projections. The black triangles indicate the direction of migration.

Under current climate, the centroid of suitable habitat was located in Guzhang County, Hunan Province (109.87° E, 28.50° N). Across future projections, the centroid showed a prevailing northwestward or southwestward displacement depending on scenario, though trajectories varied considerably (Table 3; Figure 9). Under SSP126, the centroid followed an oscillatory path, shifting southwest by about 136.55 km in the 2050s, then reversing northeast in the 2070s and 2090s, with a total path length of about 272.26 km. Under SSP245, migration was the most constrained, with a cumulative shift of about 79.19 km. SSP370 exhibited a fluctuating southwest–northeast pattern, accumulating about 191.51 km. Under SSP585, the centroid underwent the most pronounced and persistent southwestward migration, exceeding 200 km by the 2090s (Table 3).

## 4. Discussion

### 4.1. Optimization and Evaluation of the MaxEnt Model for B. officinalis

The predictive accuracy of species distribution models largely depends on parameter optimization, as default settings often produce biased predictions due to overfitting [39,59]. Through systematic parameter tuning with the ENMeval package, we identified RM = 0.5 with FC = LQ as the optimal configuration, which not only achieved a delta.AICc of 0 but also exhibited superior predictive conservatism (OR10 = 0.1511). This finding is consistent with the current consensus in the literature that lower regularization multipliers and simpler feature combinations generate more ecologically realistic predictions by controlling model complexity [15,42]. The optimized model achieved an AUC of 0.916, which was only marginally lower than the default of 0.918, while still far surpassing the ≥ 0.9 outstanding threshold defined by Swets [60]. A slight decrease in AUC following optimization but improved transferability has been reported across multiple taxa and is interpreted as a reduction in overfitting rather than a loss of predictive power [11,61]. Comparable AUC trade-offs following ENMeval optimization have been documented in other Chinese woody and medicinal plants modeled with the same framework [54,62], where modest AUC declines were accompanied by reduced overfitting and improved transferability.

Beyond AUC, the use of threshold-dependent evaluation metrics provides a more comprehensive assessment of model performance. In this study, the optimized model yielded a TSS of 0.769 ± 0.008, indicating good performance, and a Kappa of 0.417 ± 0.017, representing moderate-to-good agreement. The TSS provides a more robust measure of predictive accuracy than the AUC because it accounts for species prevalence and random agreement [63]. The moderate Kappa coefficient is expected for a widely distributed species, as niche heterogeneity often prevents perfect classification agreement between observed and predicted distributions [64,65,66]. These multi-metric evaluations collectively confirm that the optimized MaxEnt model reliably captures the key species-environment relationships governing the distribution of *B. officinalis*, providing a credible foundation for subsequent spatial predictions.

In this study, we used the 10th percentile training presence threshold for binarization, a methodologically conservative strategy that explicitly accommodates sampling noise and marginal occurrence points [39,49]. By allowing a small number of known occurrence points to fall outside the predicted suitable area, this threshold effectively reduces overprediction caused by spatial sampling bias or localized extreme microhabitats [50]. For *B. officinalis*, this choice is particularly necessary because the occurrence records were aggregated from digital herbaria, GBIF, and academic literature, sources that carry disparate degrees of spatial uncertainty [12,67]. The resulting current potential suitable area, estimated at 263.61 × 10^4^ km^2^ based on this threshold, represents a concise and ecologically reliable estimate of the potentially suitable habitat of this species. It should not be equated with the realized distribution range, as no independent field validation was conducted.

### 4.2. Principal Environmental Variables Shaping B. officinalis Suitability

Annual temperature range (Bio7, 70.6%), altitude (14.4%), and annual precipitation (Bio12, 6.0%) jointly drove over 91% of model predictions, confirming that climate and topography largely define the ecological niche of *B. officinalis*. Bio7 characterizes the seasonal variation in temperature, and its overwhelming explanatory power confirms the high sensitivity of subtropical woody plants to temperature annual range, a factor that regulates key phenological processes such as germination, flowering, and cold acclimation [3,68]. Similar dominance of temperature annual range has been reported for other subtropical woody species in China and elsewhere [69,70]. Specifically, the presence probability of *B. officinalis* exhibited a unimodal response to Bio7, with an optimal range of 16.05–29.88 °C, which is consistent with the subtropical monsoon climate characteristics of southwestern China, where moderate seasonal temperature differences are sufficient to synchronize its physiological rhythms [71,72]. Physiological studies further show that subtropical shrubs require a minimum chilling period to break bud dormancy, and that excessive seasonal thermal amplitude can disrupt this process [73]. Our estimated optimal Bio7 range is consistent with the late-winter flowering phenology of *B. officinalis*. When Bio7 exceeded 30 °C, the presence probability decreased sharply, indicating that *B. officinalis* may be unable to tolerate extreme continental seasonal temperature differences and persistent thermal stress. This finding aligns closely with its actual distribution pattern in subtropical to tropical mountainous areas of southern China and is also consistent with the biogeographic characteristics of the genus Buddleja, which is mainly distributed in warm regions of southern and southwestern China [74]. Consistent with our results, temperature-related variables, have been identified as the dominant predictors for other Chinese woody and medicinal plants modeled with optimized MaxEnt [54,62], reinforcing the generality of temperature-seasonality control on subtropical shrub distributions.

Altitude was the second most important predictor, with an optimal range of 6.53–2513.17 m. This wide vertical distribution range clearly reflects the broad adaptability of *B. officinalis* to different altitudinal gradients in the Yunnan-Guizhou Plateau and adjacent mountain systems. The unimodal response pattern, with presence probability peaking at approximately 1283 m, is closely related to the superior hydrothermal conditions and highest habitat heterogeneity observed at mid-altitudes on the Yunnan-Guizhou Plateau [75]. In this region, mid-altitude zones (approximately 1000–2000 m) often harbor the highest habitat heterogeneity and species richness [76], consistent with our estimated optimal elevation range. This region, influenced by the combined effects of the southwestern monsoon and complex topography, exhibits a unique mountain climate characterized by mild and humid conditions with no pronounced seasonal drought, providing an ideal habitat for subtropical shrubs such as *B. officinalis*. We hypothesize that the reduction in predicted suitability at low altitudes (<500 m) may partly reflect the loss of native vegetation caused by intensive agricultural activities and urban expansion, rather than climatic limitations alone [77,78]. This mechanism, however, was not directly tested in our model and warrants future field validation. A second hypothesis is that the sharp decline at high altitudes (>2500 m) may reflect physiological constraints imposed by low temperatures on growing season length and reproductive activity, consistent with the widely reported pattern that the upper altitudinal limits of woody plants are controlled by thermal conditions [3,76]. Testing this hypothesis would require physiological or demographic data beyond the scope of the present modeling approach. Notably, altitude, as a topographic proxy, not only directly reflects the temperature lapse rate but also indirectly integrates multiple environmental gradients, including soil type, slope aspect, sunshine duration, and the intensity of human disturbance. This further consolidates its importance as a composite predictor in species distribution models for topographically complex regions [79,80].

Annual precipitation (Bio12) showed a negative correlation with presence probability, with the probability monotonically decreasing once precipitation exceeded the optimal threshold. This pattern is consistent with the ecological habit of *B. officinalis*, which prefers well-drained, relatively dry, and open habitats, and supports the ecological understanding that seasonal water stress can actually favor the competitive advantage of some xerophytic or semi-xerophytic shrubs [81]. Similar preferences for moderate moisture regimes have been documented in other xerophytic shrubs, where excessive precipitation can increase competition from mesophytic species or exacerbate root anoxia [82]. Although the contribution rate of the Human Footprint Index (HFI) was relatively limited (5.7%), it aligns with growing evidence that anthropogenic pressures, including agricultural expansion, infrastructure development, and over-harvesting of wild medicinal plants, are profoundly altering the actual distribution ranges of economically valuable species [83,84]. NDVI contributed minimally (1.1%), reflecting its strong collinearity with temperature and precipitation at broad scales, where vegetation productivity is climate-driven and thus its unique contribution is reduced [34]. Furthermore, *B. officinalis* prefers relatively open, well-lit habitats, such as shrublands, forest edges, and roadsides, and its suitable areas do not necessarily correspond to densely forested regions with high vegetation cover. This further explains the low contribution rate of NDVI. The low NDVI contribution also reflects the species’ preference for open, well-lit habitats such as shrublands, forest edges, and roadsides, rather than densely forested areas with high vegetation cover [85]. These findings quantitatively delineate the multidimensional niche of *B. officinalis* for the first time, extending the generality of temperature-seasonality dominance to a subtropical medicinal shrub and providing a foundation for its conservation, germplasm utilization, and cultivation distribution.

### 4.3. Climate Change Effects on B. officinalis Suitable Ranges

The predicted core–periphery gradient, with high-suitability areas concentrated in Yunnan, Guizhou and Sichuan and declining toward the margins, is consistent with the documented distribution of *B. officinalis* [23,29] and with core–margin patterns reported for other Chinese woody and medicinal plants modeled with optimized MaxEnt [54,86]. Against this current baseline, the projected suitable area of *B. officinalis* diverges sharply under contrasting future emission pathways. Under SSP126, the overall suitable area exhibited a non-monotonic change, decreasing to 205.53 × 10^4^ km^2^ in the 2070s but rebounding to 242.55 × 10^4^ km^2^ in the 2090s. In contrast, under SSP370 and SSP585, the area of suitability showed sustained contraction, declining to 190.65 × 10^4^ km^2^ and 177.87 × 10^4^ km^2^ by the 2090s, respectively. This emission-dependent divergence is consistent with previous studies on subtropical woody species under future climate scenarios, which have reported that low-emission pathways may allow suitable areas to remain relatively stable or even expand, whereas high-emission pathways lead to severe contraction [62]. This emission-dependent pattern reveals a Bio7 (temperature annual range) threshold effect. The response curve identifies an optimal Bio7 range of 16.05–29.88 °C, with suitability declining sharply above ~30 °C. Low-emission scenarios keep projected Bio7 largely within this range, temporarily benefiting the species, whereas high-emission scenarios push Bio7 beyond ~30 °C, exceeding its thermal tolerance and causing sustained habitat contraction [3,87].

Projections under SSP585 indicate an 87.07% reduction in the high-suitability area, from 61.16 × 10^4^ km^2^ to 7.91 × 10^4^ km^2^. The magnitude of this contraction is consistent with pessimistic projections for medicinal plants globally, with multiple studies reporting severe contractions of suitable areas for species under high-emission scenarios [88,89]. Despite this marked contraction, the high-suitability core persisted across all scenarios, indicating that range loss was expressed mainly as peripheral attrition rather than wholesale core displacement, a contraction mode likewise found in future projections for comparable Chinese medicinal plants [90,91]. These findings align with macroecological predictions that species with narrow thermal niches and restricted altitudinal distributions face disproportionately high extinction risks under unchecked climate pressure [10,92].

Beyond changes in total suitable area, climate change is expected to affect *B. officinalis* habitats in several specific ways. First, rising temperatures and altered precipitation regimes may shift the species’ optimal thermal and moisture envelopes, potentially forcing populations to higher elevations or more northern latitudes. Second, increased frequency of extreme climatic events, such as heatwaves and droughts, may exceed the species’ physiological tolerance, particularly at lower elevations where populations are already near their thermal limits. Third, habitat fragmentation caused by both climate change and human activities may disrupt pollination networks and reduce gene flow among populations, further weakening adaptive capacity. These mechanisms are consistent with documented responses of other subtropical shrubs to climate change [3,93].

Under all scenarios, a general northwestward migration trend was observed for the centroid of the suitable distribution area, although the magnitude and trajectory varied considerably, from a cumulative migration of 38.47 km under the SSP245 scenario to more than 200 km under the SSP585 scenario. This directional trend is consistent with observations of poleward and upward range shifts of numerous temperate and subtropical species in the Northern Hemisphere, driven by latitudinal temperature gradients and the need to track analogous climate habitats at higher latitudes and elevations [94,95]. The southwest–northeast oscillatory migration pathways observed under the SSP126 and SSP370 scenarios may reflect decadal climate variability superimposed on the long-term warming trend, highlighting the importance of multi-period projections for capturing non-linear distributional dynamics [96]. Under the SSP126 scenario, the centroid shifted more than 120 km northeastward by the 2090s, suggesting that rapid decarbonization could facilitate the expansion of *B. officinalis* into new suitable habitats in central and eastern China, depending on the integrity of dispersal corridors and whether habitat fragmentation impedes migration [93]. However, the extensive loss of historical distribution areas, especially along the southern and low-elevation peripheries of the present range, indicates that relying solely on in situ conservation may prove insufficient.

Analysis of the spatial reorganization of suitable areas (retention, loss, and expansion) revealed that habitat retention rates decreased monotonically with increasing radiative forcing, from 90.12% under the SSP126 scenario in the 2090s to 66.97% under the SSP585 scenario. Alarmingly, over the same comparison, habitat loss rates rose from 8.76% to 32.93%, predominantly in the southern and eastern margins, which are precisely the regions projected to undergo the most severe aridification and extreme heat [82,97]. The near-zero expansion rates under high-emission scenarios (0.10–0.11%) reflect a fundamental spatial mismatch between climate velocity and habitat continuity, a phenomenon identified as a major bottleneck limiting climate-driven range shifts in sessile organisms [98,99]. From a conservation perspective, the identified retention areas, encompassing Yunnan, Guizhou, the Sichuan Basin, and Fujian, constitute priority regions for establishing nature reserves. Meanwhile, the expansion areas projected under the SSP126 scenario, such as parts of Shaanxi, Henan, and Shandong, should be actively monitored as potential sites for assisted migration or ex situ conservation [100,101]. Integrating these spatial predictions with genetic diversity assessments and population viability analyses will be essential for developing adaptive management strategies to enhance the capacity of *B. officinalis* to withstand future climate disturbances [102,103]. However, modeled suitability does not confirm actual occupancy, establishment, or population persistence. These projections should therefore be used to prioritize areas for monitoring and field validation, rather than to directly justify translocation or assisted migration.

### 4.4. Limitations and Future Directions

This study provides the first systematic assessment of *B. officinalis* habitat suitability under climate change, but several limitations should be acknowledged. First, our model relied on a single general circulation model (BCC-CSM2-MR) and four emission scenarios, which cannot fully represent inter-model uncertainty. Second, soil variables and biotic interactions were not included, although these factors may substantially constrain the realized niche of *B. officinalis*. Third, occurrence records compiled from herbaria, GBIF, and the literature may carry sampling bias, and spatial filtering at 2.5° × 2.5° may not fully eliminate it. Fourth, our analysis assumed static species–environment relationships, whereas species may respond to climate change through dispersal, phenotypic plasticity, or evolutionary adaptation. Fifth, future projections were driven only by climatic variables, while NDVI and the Human Footprint Index were held constant, potentially overlooking land-cover and anthropogenic dynamics. Finally, the predicted suitable areas represent a climatically permissible envelope rather than the realized distribution, and no independent field validation was conducted.

Future research should address these limitations correspondingly. First, multi-model ensembles should be employed to bracket inter-model uncertainty and to identify potential refugia and areas of consistent habitat loss. Second, soil properties, population genetics, and species interactions should be incorporated to refine niche estimates and improve the realism of predicted distributions. Third, field validation and long-term monitoring would help reduce sampling bias and detect early climate-driven shifts in *B. officinalis* populations. Fourth, dynamic modeling approaches that account for dispersal, phenotypic plasticity, and evolutionary adaptation should be explored to move beyond static equilibrium assumptions. Fifth, dynamic projections of land cover and human footprint should be integrated to capture the combined effects of climate and anthropogenic change. Accordingly, the areas identified in this study should be regarded as candidate regions for further investigation rather than operational conservation or translocation targets.

## 5. Conclusions

This study provides the first systematic assessment of *B. officinalis* habitat suitability under climate change across China using an ENMeval-optimized MaxEnt model. Temperature annual range (Bio7, 70.6%), altitude (14.4%), and annual precipitation (Bio12, 6.0%) were the dominant predictors, jointly explaining over 91% of the species’ distribution. Currently, high-suitability areas cover 61.16 × 10^4^ km^2^, concentrated in Yunnan, Guizhou, Sichuan, southern Gansu, and southeastern Fujian. Under future climates, suitable habitat shows clear emission-dependent divergence: under SSP126, it fluctuates but partially recovers by the 2090s, whereas under SSP585, the high-suitability area declines by 87.07% to 7.91 × 10^4^ km^2^ in the 2050s, with the distributional centroid shifting more than 200 km northwestward. Retention zones in Yunnan, Guizhou, and Sichuan represent priority areas for conservation planning, while potential expansion areas in Shaanxi, Henan, and Shandong warrant close monitoring. These projections identify candidate areas for further investigation. Given the single-GCM approach and the absence of independent field validation, the results should not be used to support operational translocation or assisted migration measures without additional empirical evidence. These findings provide a quantitative basis for identifying priority areas for monitoring and field validation, and inform future research on cultivation suitability and wild resource conservation of *B. officinalis*.

## Figures and Tables

**Table 1:** Area and proportion of suitable habitat classes for *B. officinalis* under current and future climate scenarios.

Scenario-Period	Area (10^4^ km^2^)				Percentage (%)			
	Total	Low	Medium	High	Total	Low	Medium	High
SSP126-2050s	263.61	115.40	87.06	61.16	27.46	12.02	9.07	6.37
SSP126-2070s	207.41	118.82	65.52	23.07	21.61	12.38	6.83	2.40
SSP126-2090s	205.53	129.86	60.21	15.46	21.41	13.53	6.27	1.61
SSP245-2050s	242.55	155.57	68.24	18.74	25.27	16.21	7.11	1.95
SSP245-2070s	247.88	151.60	74.38	21.90	25.82	15.79	7.75	2.28
SSP245-2090s	242.81	146.42	75.09	21.29	25.29	15.25	7.82	2.22
SSP370-2050s	233.19	143.27	70.39	19.54	24.29	14.92	7.33	2.04
SSP370-2070s	227.11	132.66	71.56	22.89	23.66	13.82	7.45	2.38
SSP370-2090s	224.55	145.88	64.92	13.76	23.39	15.20	6.76	1.43
SSP585-2050s	190.65	130.69	52.02	7.94	19.86	13.61	5.42	0.83
SSP585-2070s	232.32	138.40	72.27	21.65	24.20	14.42	7.53	2.26
SSP585-2090s	211.13	138.14	60.35	12.64	21.99	14.39	6.29	1.32

**Table 2:** Temporal variations in the suitable range of *B. officinalis* under alternative future climate projections.

Scenario-Period	Area (10^4^ km^2^)			Rate of Change (%)		
	Stability	Contraction	Expansion	Stability	Contraction	Expansion
SSP126-2050s	205.68	58.65	0.28	77.73	22.16	0.11
SSP126-2070s	204.88	59.41	0.24	77.45	22.46	0.09
SSP126-2090s	240.91	23.42	2.98	90.12	8.76	1.11
SSP245-2050s	241.16	22.70	7.10	89.00	8.38	2.62
SSP245-2070s	236.43	27.54	6.55	87.40	10.18	2.42
SSP245-2090s	225.61	38.70	7.69	82.94	14.23	2.83
SSP370-2050s	224.38	39.98	2.27	84.16	14.99	0.85
SSP370-2070s	222.56	41.76	2.18	83.51	15.67	0.82
SSP370-2090s	189.60	74.72	0.27	71.66	28.24	0.10
SSP585-2050s	230.38	33.94	2.01	86.50	12.74	0.75
SSP585-2070s	209.54	54.23	1.48	79.00	20.45	0.56
SSP585-2090s	176.89	86.97	0.29	66.97	32.93	0.11

**Table 3:** Centroid shifts of suitable habitat for *B. officinalis* under future climate scenarios. Coordinates refer to the geometric centroid of predicted suitable habitat at 2.5 arc-minute resolution, not actual population locations. Distances are approximate, measured from the previous time slice using the Vincenty formula. SW = southwest, NE = northeast, NW = northwest.

Scenario-Period	Longitude (°)	Latitude (°)	Direction	Distance from Previous Centroid (km)
Current	109.87	28.50	—	—
SSP126-2050s	108.60	28.00	SW	136.55
SSP126-2070s	108.75	28.03	NE	15.32
SSP126-2090s	109.80	28.58	NE	120.39
SSP245-2050s	109.67	28.80	NW	38.47
SSP245-2070s	109.50	28.77	SW	16.12
SSP245-2090s	109.25	28.80	NW	24.6
SSP370-2050s	109.00	28.40	SW	85.36
SSP370-2070s	109.12	28.57	NE	21.75
SSP370-2090s	108.33	28.23	SW	84.4
SSP585-2050s	109.25	28.47	SW	59.59
SSP585-2070s	108.85	28.40	SW	39.73
SSP585-2090s	107.95	27.78	SW	112.59

## Data Availability

The original contributions presented in this study are included in the article/Supplementary Material. Further inquiries can be directed to the corresponding author.

## Supplementary Materials

The following supporting information can be downloaded at: https://www.mdpi.com/article/10.3390/biology15211984/s1. Table S1. Twenty-four environmental variables used in this study.
