# Supplementary material for: Spatiotemporal Dynamics of Buddleja officinalis Maxim. Habitat Suitability Under Climate Change in China
Source: Biology (Basel). 2026 Nov 2;15(21):1984. doi: 10.3390/biology15211984 (PMC13761709; doi:10.3390/biology15211984)
Supplement: Supplementary file 1 [file biology-15-01984-s001.zip › biology-4582123-supplementary.pdf]

Supplementary materials

Spatiotemporal Dynamics of *Buddleja officinalis* Maxim. Habitat Suitability Under Climate Change in China

Table S1. Twenty-four environmental variables used in this study.

Category	Abbreviation	Environmental variables	Units	Range	Percent contribution	Weather for Modeling
Bioclimatic	Bio1	Annual mean temperature	°C	2.05 – 22.91	0.3	No
	Bio2	Mean diurnal range (Mean of monthly)	°C	6.04 – 16.09	1	No
	Bio3	Isothermality (Bio2/Bio7) (× 100)		22.75 – 53.80	1.1	No
	Bio4	Standard deviation of temperature seasonality		317.59 – 1014.06	2.2	No
	Bio5	Max temperature of warmest month	°C	13.52 – 33.61	0.5	No
	Bio6	Min temperature of coldest month	°C	-12.73 – 10.70	26.6	No
	**Bio7**	**Temperature annual range (Bio5-Bio6)**	**°C**	**19.16 – 37.28**	**4**	**Yes**
	Bio8	Mean temperature of wettest quarter	°C	8.97 – 28.96	0.8	No
	Bio9	Mean temperature of driest quarter	°C	-4.12 – 19.68	0.6	No
	Bio10	Mean temperature of warmest quarter	°C	8.97 – 28.96	0.7	No
	Bio11	Mean temperature of coldest quarter	°C	-5.41 – 18.14	1.6	No
	**Bio12**	**Annual precipitation**	**mm**	**558.00 – 2019.00**	**40.8**	**Yes**
	Bio13	Precipitation of wettest month	mm	107.00 – 400.00	0	No
	Bio14	Precipitation of driest month	mm	1.00 – 48.00	0.4	No
	**Bio15**	**Variation of precipitation seasonality**		**45.95 – 108.43**	**2.8**	**Yes**
	Bio16	Precipitation of wettest quarter	mm	292.00 – 947.00	0	No
	Bio17	Precipitation of driest quarter	mm	8.00 – 193.00	0.1	No
	Bio18	Precipitation of warmest quarter	mm	281.00 – 943.00	0.5	No
	Bio19	Precipitation of coldest quarter	mm	8.00 – 207.00	0.6	No
Topographic	**Altitude**	**Altitude**	**m**	**4.00 – 4010.00**	**7.3**	**Yes**
	**Aspect**	**Aspect**	**°**	**1.18 – 359.52**	**0.5**	**Yes**
	**Slope**	**Slope**	**°**	**0.02 – 9.47**	**2.3**	**Yes**
Vegetation	**NDVI**	**Normalized difference vegetation index**		**0.18 – 0.82**	**0.1**	**Yes**
Human	**HFI**	**Human footprint index**		**1.14 – 50.00**	**5.3**	**Yes**
